# Protective Buffering and Individual and Relational Adjustment Following Hematopoietic Stem Cell Transplantation: A Dyadic Daily-Diary Study

**DOI:** 10.3389/fpsyg.2019.02195

**Published:** 2019-09-24

**Authors:** Aleksandra Kroemeke, Małgorzata Sobczyk-Kruszelnicka

**Affiliations:** ^1^Department of Psychology, SWPS University of Social Sciences and Humanities, Warsaw, Poland; ^2^Maria Skłodowska-Curie—Oncology Center, Gliwice, Poland

**Keywords:** social support, relationship quality, relationship stress, affect, cancer, hematopoietic stem cell transplantation, dyadic study, daily-diary study

## Abstract

**Background:**

Supportive communication (e.g., protective buffering, PB) may impact individual and relational adjustment in patients following hematopoietic stem cell transplantation (HSCT) and their caregivers. Previous studies revealed that PB (i.e., hiding one’s concerns and denying one’s worries) has mixed effects, namely it is beneficial, costly or unrelated to dyadic adjustment. This study aimed to verify these findings by addressing some unresolved issues, i.e., examining (1) both individual and relational as well as both positive and negative indicators of adjustment, (2) the effect of within-dyad congruence (i.e., complementarity/similarity) in PB, and (3) within-dyad causal associations between PB and adjustment.

**Methods:**

Two hundred patients (following first autologous or allogeneic HSCT) and their caregivers independently completed measures of daily PB, relationship satisfaction, relationship stress, and positive affect (PA) and negative affect (NA) for 28 consecutive evenings after discharge of patients.

**Findings:**

For both patients and caregivers, the results showed a same-day association between daily PB and individual (positive and negative) and relational (positive and negative) adjustment indicators showing the advantage of PB. In terms of the dyad congruence, complementarity (one partner high and the other low) in daily PB was related to higher same-day relationship satisfaction for both patients and caregivers and lower same-day relationship stress in caregivers. The benefits from similarity (both patient and caregiver high or low in PB) had delayed effects, although only in patients. As far as the causal associations were concerned, day-to-day changes in PB preceded changes in daily adjustment. In caregivers, reverse causality was found, i.e., changes in adjustment predicted next-day changes in support.

**Discussion:**

Contrary to previous studies, daily PB has a rather beneficial effect in dyads following HSCT. Patients seemed to have benefited the most from the similarity in daily PB fluctuation, while caregivers profited from complementarity. Causal associations between PB and adjustment within-dyad were also different. The findings may add to a better understanding of PB-adjustment relationship in dyads facing HSCT.

## Introduction

**FIGURE 1:**
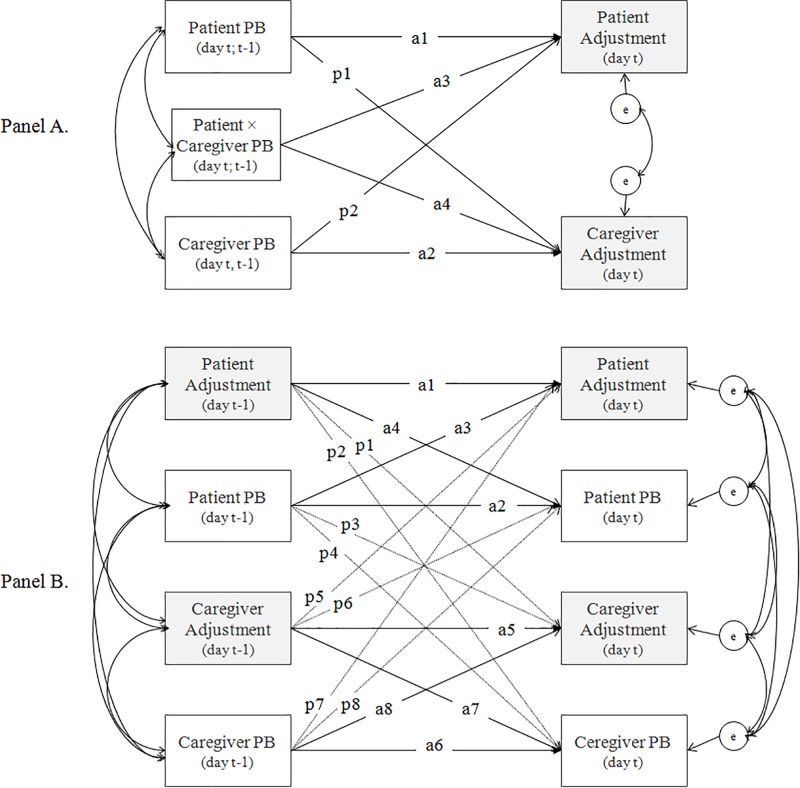
Conceptual model—the dyadic relationship between the protective buffering (PB) and adjustment indicators. **(A)** The Actor-Partner-Interdependence Model (APIM) of the concurrent (same-day, day *t*) and lagged (previous-day, day *t* – 1) effects of daily PB on daily adjustment in dyads (intrapersonal effects = paths a1 to a2; interpersonal effects = paths p1 to p2) with the interaction (paths a3 and a4) of PB between dyad members. **(B)** The autoregressive cross-lagged model of daily PB and adjustment indicators (intrapersonal effects = paths a1–a8; interpersonal effects = p1–p8).

Adjustment to cancer or other chronic diseases is a challenging process which involves not only the patient but also his/her surroundings. According to the most common definition of stress (Lazarus and Folkman, 1984), disease and its treatment may be perceived as a stressful situation. Based on Lazarus and Folkman (1984) stress and coping model, adjustment to stressful circumstances is a dynamic and time-varying process that requires coping efforts from an individual and availability of their personal and social resources. Indeed, any chronic disease is a shared stressor, as it affects patient’s family, and therefore needs to be considered from the dyadic perspective (Revenson and DeLongis, 2011). Methodologically, the Actor-Partner-Interdependence-Model (APIM) describes the scheme of dyadic relationship (Kenny et al., 2006). APIM differentiates between *intrapersonal effects* (i.e., an individual’s effect of the predictor variable on the same individual’s score of the outcome variable; see Figure 1A, paths a1 and a2) and *interpersonal effects* (i.e., the effect of one person’s predictor variable on the other person’s outcome variable; see Figure 1A, paths p1 and p2). Referring to Lazarus and Folkman (1984) framework to stress, the ways of coping, resources (e.g., social support), and adjustment indicators in one dyad member may impact the same indicators and mechanisms in the other.

From the dyadic perspective, the social support *patterns* in dyads facing chronic disease are of particular interest (Revenson and DeLongis, 2011). One of the social support patterns that may impact the risk for cancer adjustment in the patient and their family is protective buffering (PB). Cancer-related PB is defined as a “hiding one’s concerns, denying one’s worries, concealing discouraging information, preventing the patient from thinking about the cancer, and yielding” (Hagedoorn et al., 2000, p. 275). Although PB is classified as a provided support or supportive communication (Revenson and DeLongis, 2011) and is a part of social support questionnaires (Ways of Giving Support Questionnaire, Buunk et al., 1996; Berliner Social Support Scales, Schulz and Schwarzer, 2003), others treat it as coping efforts (Coyne and Smith, 1994). Here, we adopted the first concept, however, we referred to both in the literature review.

### Protective Buffering and Adjustment in Dyadic Research

The main purpose of PB in the disease context is to protect the close person and the relationship against disease-related stress. Potentially, PB appears to have a beneficial effect, especially for a “protected” person. Such a person may experience a lower level of stress in this situation when they are unaware of certain aspects of the situation and, above all, of the partner’s fears and anxieties. The beneficial effect of PB for the “protective” person may result from experiencing a sense of control over the situation and occur through the processes of transmission in the dyad, i.e., mood contagion (Neumann and Strack, 2000). So far, however, only a few studies have confirmed this view. Coyne and Smith (1994) noted higher spouse-reported PB associated with higher post-myocardial infarction self-efficacy of patients. Outside the context of disease, Finkenauer and Hazam (2000) found a positive effect of secrecy (information that one partner actively and consciously withholds from the other partner) on marital satisfaction in a “protective” person; but they noted a negative function of perceived secrecy for a “protected” person. However, these studies did not apply a fully reciprocal APIM allowing for estimation of both intra- and inter-personal effects. Several experimental studies (outside the dyadic perspective) have also proved that suppression of the expression of emotion is adaptive at least under certain conditions (Bonanno et al., 2004; Liverant et al., 2008; Roberts et al., 2008). Applying APIM in healthy and ill dyads, Badr (2004) found the benefits from congruence in PB between dyad members. Complementarity, not similarity, in the PB level was related to greater marital satisfaction in dyads.

The costs associated with PB are more often emphasized, especially for the person who uses it. Suppressing and hiding one’s emotions, especially negative ones, disturb the functioning of the individual in many areas: affective, cognitive, social, and physiological (see Gross, 2002; Bonanno and Burton, 2013 for review). Persistent tension associated with suppression and emotional protection of the close person in a difficult situation will be potentially unfavorable for the “protective” person. However, through the already discussed transmission mechanisms, it may also affect the “protected” person. This view is supported by a growing number of research (mainly in the cancer setting) that link cross-sectionally higher PB with lower relationship satisfaction (Coyne and Smith, 1991; Langer et al., 2009), higher distress (Coyne and Smith, 1991; Suls et al., 1997; Manne et al., 2010) and lower intimacy (Manne et al., 2010) in both dyad members. Longitudinally, higher PB was linked to higher distress in post-myocardial infarction patients (Coyne and Smith, 1991; Suls et al., 1997), while in breast cancer caregivers, higher initial distress predicted more PB over six and 12 months on average (Hinnen et al., 2007). Similarly, a negative effect of PB was found in relation to interpersonal effects. Higher spouse-reported PB predicted higher breast cancer patient-reported distress at three and 9 months after diagnosis (Hinnen et al., 2009), as well as higher depressive symptoms in cardiac patients at 6-month follow-up (Vilchinsky et al., 2011).

Other studies revealed that PB was unrelated to dyadic well-being in cross-sectional (Kuijer et al., 2000; Manne et al., 2014) and longitudinal studies (Langer et al., 2009). A relatively large part of research on the role of PB is related to the received PB. Higher perception of PB as provided by the partner was related to lower marital satisfaction in cancer (Hagedoorn et al., 2000) and diabetes patients (Schokker et al., 2010). Longitudinally, it negatively impacted health-related quality of life of asthma and diabetes patients (de Ridder et al., 2005) and marital satisfaction in colon and rectal cancer patients (Hagedoorn et al., 2011) as well as breast cancer women (Hinnen et al., 2008). Contrary, Vilchinsky et al. (2011) reported higher patient-perceived PB as provided by the spouse linked to lower depressive symptoms in cardiac patients 6 months later.

### Open Issues Related to the Association Between Protective Buffering and Adjustment in Dyadic Research

#### Adjustment Levels (Individual/Relational) and Their Valence (Positive/Negative)

Although research on PB within dyads has led to important findings, their results are ambiguous and a number of unresolved issues still remain. First, research on PB in dyads focused mostly on relationship satisfaction or depressive symptoms and distress (see section “Protective Buffering and Adjustment in Dyadic Research”). Therefore, our knowledge on the role and mechanisms of PB in dyads is limited to the positive relational indicator of adjustment and the negative individual adjustment. In contrast, only one study has examined positive individual-level adjustment (de Ridder et al., 2005). Many studies showed that positive and negative states are not simple opposites and the mechanisms that produce them are distinct (Larsen and McGraw, 2011). Therefore, it can be expected that relations connecting PB with respectively, positive and negative adjustment indicators will also be specific. Moreover, the effect of PB on individual and relational adjustment should be specific. Since this action is to be focused on the protection of the relationship with the partner and the partner himself/herself, it should be beneficial for both partners at the relational level. However, possible costs related to this form of support should occur at the individual level, mainly by intraindividual effects. Although previous studies did not confirm these hypotheses, they are limited to selected positive or negative relational or individual adjustment indicators as already indicated. In addition, they rarely simultaneously consider more than one aspect of adjustment and fully reciprocal dyadic design according to APIM.

#### Within-Person(Dyad) Variability

Second, previous studies focused on between-person differences in the relationship between PB and adjustment (see section “Protective Buffering and Adjustment in Dyadic Research”). Indeed, all processes vary across both participants (between-person approach) and time (within-person approach) and regularities found in one of these approaches are unlikely to mirror the obtained regularities in the other (Bolger and Laurenceau, 2013). This allows to suppose that knowledge on the relationship between PB and adaptation obtained in studies related to the between-person approach will not necessarily reflect the within-individual (dyad) processes. Intensive longitudinal design (ILD) allows to examine within-person change. We found only two reports that used ILD to test the relationship between fluctuation of PB and adjustment in dyads. Using a 12-day diary and APIM among 58 heart disease patients and their spouses, Butler et al. (2004) found that higher than usual daily PB in patients and their spouses was associated with concurrent (same-day) lower affect balance in participants and their partners. In cancer setting, daily holding back from expressing feelings was related to lower concurrent (not lagged, i.e., next-day) relationship satisfaction in both patients and spouses (Langer et al., 2018). These studies seem to support the hypothesis on unfavorable effects of daily PB for individual well-being in terms of both intra- and inter-individual effects.

#### Reciprocal (Causal) Dependencies

Finally, previous studies examined mostly cross-sectional and less frequently longitudinal relationships between PB and adjustment (see section “Protective Buffering and Adjustment in Dyadic Research”), assuming the direction from PB to adjustment. Although this is part of schematic relations described in the Lazarus and Folkman (1984) stress and coping model, it does not consider dynamic or reciprocal relationships between variables involved in the coping process. Researchers often indicate in the limitation sections that the direction of influences between PB and adjustment may be reverse. However, only two studies have examined it (Hinnen et al., 2007; Langer et al., 2018). Increased suppression and hiding emotions can be the result (and not the cause) of experiencing greater distress and lower relationship satisfaction (through reluctance to get involved in an unsatisfying relationship), particularly in the case when day-to-day fluctuation of these processes is considered.

### The Present Study

The present study attempted to address the already described unresolved issues by (1) including opposite valences (positive/negative) of two levels of adjustment (individual/relational), (2) considering the day-to-day fluctuation in PB-adjustment relationship, and (3) examining the reciprocal associations between PB and adjustment in dyads. The study aimed to examine the association between PB and positive affect (PA), negative affect (NA), relationship satisfaction, and relationship stress in dyads following hematopoietic stem cell transplantation (HSCT) using the daily process methodology. HSCT is one of the most aggressive but also effective forms of cancer treatment. It is based on destruction of the hematopoietic system of the patient as a result of intense radio and/or chemotherapy. Next, autologous or allogeneic stem cell transplantation is performed with the aim to restore hematopoietic and immune systems. The procedure is preceded by preparation for the transplant (including cell collection in autologous HSCT or waiting for a donor in allogeneic HSCT) followed by a period of patient isolation lasting several weeks. In the out-patient period, patients still experience various adverse symptoms (e.g., loss of appetite, mouth problems or fatigue). They also have to follow the rules related to hygiene, diet, medicine intake, frequent follow-up visits, and contacts with other people, for which caregivers are most responsible. This situation seems to favor PB. Patients who do not want to worry caregivers can hide their poor physical/mental condition and the complaints they experience. Caregivers, in turn, are usually afraid of their partner’s life. Therefore, they can hide their worries related to them. In addition, they may want to protect patients from other problems that are not related to the disease (e.g., work- or child-related problems). In particular, this study assessed the effect of within-dyad congruence in daily PB and the within-dyad reciprocal dependency in the relationship of daily PB and adjustment indicators.

To verify the effect of congruence in daily PB on adjustment in our dyads, we examined the conceptual model shown in Figure 1A. According to Revenson (1994), dyads maximize the congruence between behaviors of the partners to achieve the best adaptation. Congruence can involve either similarity (both dyad members have a similar level of a certain variable, low or high) or complementarity of partners’ behavior (one dyad member has a higher level of a certain variable than the other; Revenson, 1994). Complementarity was found to be more effective for avoidance or emotion-focused behaviors (Revenson and DeLongis, 2011), such as PB (Badr, 2004). Therefore we anticipated complementarity (not similarity) in PB fluctuation to be related to better dyadic individual and relation adjustment in our sample (Hypothesis 1). We operationalized congruence as an intrapersonal interaction effect, i.e., patient daily PB × caregiver daily PB (see Figure 1A, paths a3 and a4), which allowed us to test the effect of similarity (high or low daily PB in both parties) or complementarity (one partner high and the other partner low in daily PB). In addition, based on PB function in dyads, we expected the benefits of daily PB for relational adjustment and detrimental effects for individual adjustment (Hypothesis 2). We tested the concurrent (same-day; day *t*) and lagged (next-day; day *t* − 1 to day *t*) effects to separate the correlation effects from the short-time predictions.

To examine the reciprocal dependency (causality) in PB and adjustment fluctuation in our dyads, the model presented in Figure 1B was tested. The model consists of autoregressive (paths a1, a2, a5, a6, and p1, p4, p5, p8) and cross-lagged effects (paths a3, a4, a7, a8, and p2, p3, p6, p7), both of which may relate to intra- and inter-personal effects (paths denoted as *a* and *p*, respectively). Autoregressive effect indicates to what extent each variable (PB and adjustment indicators for both patients and caregivers) is predictive of itself over time (controlling for the partner’s score) (Bringmann et al., 2018). Its positive value indicates that the process is resistant to change. Therefore this parameter is also known as inertia or regulatory weakness (De Haan-Rietdijk et al., 2016; Bringmann et al., 2018; Hamaker et al., 2018). The cross-lagged parameters reflect a predictive relationship, i.e., the direction and strength of the effect of one variable on the other (PB on next-day adjustment or vice versa; within person and dyad) controlling for the autoregressive effects (De Haan-Rietdijk et al., 2016; Bringmann et al., 2018; Hamaker et al., 2018). Due to the lack of available premises, we did not formulate hypotheses on the direction of influence in this relationship. However, considering that behavioral and affective processes may be less determined by each other and more by regulatory weakness (Kuppens et al., 2010), we expected autoregressive effects to be stronger than cross-regression effects in both patients and caregivers (Hypothesis 3).

## Materials and Methods

### Participants and Procedure

The study was conducted in accordance with the recommendations of the SWPS University of Social Sciences and Humanities Ethics Committee and the University Ethics Committee approved the protocol (decision no. 24/2014). All participants gave written informed consent in accordance with the Declaration of Helsinki. Participation in the study was voluntary. The study is part of a larger project dedicated to the complexity and dynamics of the coping process in dyads following HSCT.

Recruitment occurred in a single clinic following elective admission due to HSCT. The patient inclusion criteria in the study were as follows: (a) admission to the first autologous or allogeneic HSCT, (b) age ≥ 18 years, (c) no history of other major disabling medical or psychiatric conditions, and (d) efficiency in reading and writing. Eligible patients who gave written informed consent (*N* = 285) filled in the baseline assessment (demographic items), while the clinical data were obtained from medical records. The contact with a caregiver was established by phone. The inclusion criteria for caregivers were as follows: (a) age ≥ 18 years, (b) no history of other major disabling medical or psychiatric conditions, (c) close contact and patient care during the outpatient recovery period following HSCT, and (d) efficiency in reading and writing. Two hundred fifty-two caregivers consented to participate. Demographic data of caregivers and written informed consent were collected on the first day of the diary entries. The daily study started on the first day after patient hospital discharge. All participants were instructed in detail how to complete the diary, especially in terms of timing (each evening for 28 consecutive days) and independent diary completion. Each diary completion took approximately 6–8 min. All dyads completed self-report web-based (12.5%) or paper-and-pencil (87.5%) diaries (paper versions were returned after the 28-day assessment period). The study participants also received a short text message each evening as a reminder to fill in a diary and were called three times during the 28-day period to address any difficulties.

Of 252 dyads who consented to participate, six patients were not eligible for HSCT, 17 patients died during hospitalization, three dyads withdrew their consent, 17 did not return filled-in diaries after the 28-day period, and nine dyads completed fewer than five diary days. Sample attrition analyses indicated that allogeneic HSCT was associated with an increased likelihood of belonging to the non-completer group as compared to autologous HSCT (*B* = 0.98, SE = 0.36, *p* < 0.001, OR = 2.68).

The final sample consisted of 200 patient–caregiver dyads. Most participants were middle aged (*M* = 47.85 years, SD = 13.48, range = 19–68 and *M* = 47.38 years, SD = 13.11, range = 18–73, for patients and caregivers, respectively) and had at least a secondary education (*M* = 14.18 years of education, SD = 3.32, range = 7–28 and *M* = 14.07 years of education, SD = 3.29, range = 7–25, for patients and caregivers, respectively). Patients were mostly male (57%), not working (63%), diagnosed with lymphomas (48%; 17.5% leukemias and other myeloid neoplasms; 31% multiple myeloma; 3.5% other cancer types) who underwent autologous HSCT (74%; 26% allogeneic HSCT) and high-intensity conditioning (97%; 88.5% of autologous HSCT recipients). The mean time from diagnosis was 21.89 months (SD = 24.07, range = 3–180) and the mean time from HSCT to discharge was 18.51 days (SD = 9.32, range = 10–91; for autologous HSCT recipients: *M* = 14.45 days, SD = 3.52, range = 10–33; for allogeneic HSCT recipients: *M* = 30.08 days, SD = 10.91, range = 17–91). Caregivers were mostly female (70.5%) and employed (61.5%). Most dyads consisted of spouses or romantic partners (77.5%; 11% parent/child dyads; 8% child/parent dyads; 3% siblings dyads; 0.5% other). The mean duration of the relationship was 25.34 years (SD = 12.26, range = 1–56).

### Measures

#### Daily Protective Buffering

The participants completed three items (“I avoided everything that could upset him/her”; “I showed strength in his/her presence”; “I did not let him/her notice how bad and depressed I really felt”) from the Berlin Social Support Scale (BSSS, Schulz and Schwarzer, 2003) adapted to daily procedure. They rated the extent of PB on a particular day using a 4-point scale ranging from 1 (*not at all*) to 4 (*very strongly*). Higher scores indicated greater daily PB as reported by individuals (total daily score: 3–12). Within-person reliabilities (coefficient omega) were 0.71 for both dyad members, while between-person reliabilities (coefficient omega) were 0.91 for patients and 0.88 for caregivers.

#### Daily Relationship Satisfaction

The participants completed a three-item Kansas Marital Satisfaction Scale (Schumm et al., 1986) adapted to daily approach. They assessed how satisfied they were (a) with their study partner “today,” (b) with their contacts with the study partner “today,” and (c) with their relationship with their study partner “today,” using a 5-point scale ranging from 1 (*not at all*) to 5 (*very strongly*). Higher scores indicated greater daily relationship satisfaction as reported by individuals (total daily score: 3–15). Within-person reliabilities were 0.71 for both dyad members, while between-person reliabilities were 0.92 for patients and 0.98 for caregivers.

#### Daily Relationship Stress

The participants used a 5-point scale ranging from 1 (*not at all*) to 5 (*very strongly*) to answer the question “how stressful was my relationship with my study partner today?”. Higher scores indicated greater daily relationship stress as reported by individuals (total daily score: 1–5).

#### Daily Positive and Negative Affect

The participants rated how they felt on a particular day using a 7-point scale ranging from 1 (*not at all*) to 7 (*very strongly*). They assessed six PA items (happy, enthusiastic, content, pleasant, excited, relaxed; within-person reliability was 0.89, between-person reliability was 0.94, for both dyad members) and six NA items (unhappy, irritable, bored, sad, nervous, sluggish; within-person reliability was 0.89 for both dyad members, between-person reliabilities were 0.90 and 0.94 for patients and caregivers, respectively). Adjectives reflected the neutral, as well as low *versus* high affect arousal according to the Circumplex Model of Emotion by Larsen and Diener (1992). Higher scores indicated greater daily PA or NA as reported by individuals (total daily score per scale: 6–42).

### Statistical Analysis

Mplus statistical package version 8 (Muthén and Muthén, 1998) was used to conduct all analyses. To examine the conceptual model shown in Figure 1A, we applied multilevel structural equation modeling (MSEM) using the code developed by Laurenceau and Bolger (2013). We estimated random effects for pairs of intercepts, intrapersonal effects, interpersonal effects and interaction effects for both patients and caregivers (see Figure 1A). Because PB varied both between- and within-person and because this study focused on within-person change, PB were split into between-person (stable between-person mean for each person across all their diary days) and within-person (the deviation from the between-person mean) products for both patients and caregivers. In the lagged model, daily adjustment (in day *t*) was predicted by previous-day (*t* − 1) PB, controlled for *t* − 1 respective adjustment indicator. Significant effects of patient–caregiver interaction were graphed and probed with simple slope analyses. Unstandardized coefficients were used to plot slopes for the association between daily PB of one dyad member and adjustment at one standard deviation below and above the mean of PB of the other dyads member (Jaccard et al., 1990). Linear time trends were controlled in the analyses and centered on the middle time point. We used maximum likelihood as an estimator. In MSEM, missing data are handled within the analyzed model using a full information maximum likelihood (FIML), which is one of the best tools for missing data management (Enders and Bandalos, 2001; Newman, 2014). The Log likelihood, the Akaike information criterion (AIC), and the sample-size adjusted Bayesian information criterion (SSABIC) determined the model fit. To rule out confounds, all MSEM models were repeated with covariates (demographics and clinical variables, which were significantly related to the intercepts and/or slopes of participant’s daily adjustment in preliminary analyses, i.e., patient’s and caregiver’s sex, age, and education; patient employment; relationship duration; type of transplant and conditioning).

To examine the conceptual model shown in Figure 1B, dynamic structural equation modeling (DSEM; Asparouhov et al., 2018) was applied using multilevel vector autoregressive modeling (VAR(1); Hamaker et al., 2018). The code developed by Hamaker (2017) was used. A multilevel VAR(1) model consists of a set of regression equations, in which each of the endogenous variables was regressed on its own lagged values (autoregression) and the lagged values of the other variables (crossregression) for each individual in the dyad (intraindividual effects) and across dyad members (interindividual effects; see Figure 1B). We estimated separate models for each adjustment indicator. PB and adjustment indicators for both patients and caregivers were decomposed into within-person and between-person products (as in MSEM). To compare the strength of cross-lagged associations, within standardization (i.e., standardization using within-person variance) was preformed (Schuurman et al., 2016). DSEM used Bayesian estimator based on the Markov chain Monte Carlo algorithm (Asparouhov et al., 2018). Parameter estimates were obtained from posterior distribution (based on non-informative priors), while significance of individual parameters were evaluated based on the credible intervals (CIs) of these posterior distribution. We used the Mplus default priors (mean = 0, variance = 10^10^); the number of iterations was 5000. In DSEM, missing data are sampled from the conditional posterior and are estimated as other model parameters (Hamaker et al., 2018). In Bayesian analysis, the deviance information criterion (DIC) determined the model fit (Asparouhov et al., 2018). Model convergence was checked by inspecting the trace plots regarding irregularities. To rule out confounds, all VAR(1) models were repeated with significant covariates.

## Results

### Preliminary Analyses

Missing data analysis showed missing values were less than 11% (across all days and participants) and were slightly higher for caregivers (8.4% relationship stress, 9.1% relationship satisfaction, 9.3% NA, 10% PA, 10.9% PB) than patients (7.2% relationship stress, 8.3% relationship satisfaction, 7.9% NA, 8% PA, 9.9% PB). A total of 141 out of 200 dyads (70.5%) completed at least 26/28 daily diaries (83% of patients, *M* = 26.21 days, SD = 4.47, range = 5–28; and 75% of caregivers, *M* = 25.68 days, SD = 4.45, range = 6–28). The missing pattern analysis indicated significant differences in the type of transplant between the participants who fully and partially completed the diary (autologous HSCT was associated with full diary entries; χ^2^ = 99.33, *p* < 0.001) and daily relationship stress of patients (a decrease in patient daily relationship stress was associated with full diary entries; *Est*. = 0.01, SE = 0.003, *p* < 0.05). The final analysis dataset consisted of 4740 daily reports in MSEM and 5600 daily reports in DSEM.

Descriptive statistics and correlations of daily PB and adjustment are presented in Table 1. Most of within dyad member correlation coefficients indicated a small-to-moderate (0.10 ≤ *r* ≤ 0.30) effects based on Cohen’s (1988) criteria, with stronger associations for between-person effects, especially in caregivers. Most of cross dyad member correlation coefficients indicated a small (*r* ≤ 0.10) or small-to-moderate (*r* ≤ 0.30) effects, with stronger associations for between-person effects. The critical *p*-value for the correlation analysis was *p* < 0.05.

**TABLE 1:** Between-person descriptive statistics and between- (above the diagonal) and within-person (below the diagonal) correlations (*N* = 200 dyads).

	***M***	***SD***	**1**	**2**	**3**	**4**	**5**	**6**	**7**	**8**	**9**	**10**
*Patients*												
1. Protective buffering	7.49	2.63	1	0.21^∗∗^	0.03^∗^	0.12^∗∗^	0.04^∗∗^	0.18^∗∗^	0.02	0.00	–0.02	–0.05^∗∗^
2. Relationship satisfaction	12.67	2.59	0.19^∗∗^	1	–0.38^∗∗^	0.25^∗∗^	–0.15^∗∗^	0.18^∗∗^	0.34^∗∗^	–0.22^∗∗^	0.16^∗∗^	–0.15^∗∗^
3. Relationship stress	1.34	0.69	–0.06^∗∗^	–0.26^∗∗^	1	–0.16^∗∗^	0.42^∗∗^	–0.02	–0.22^∗∗^	0.26^∗∗^	–0.15^∗∗^	0.23^∗∗^
4. Positive affect	14.75	4.73	0.10^∗∗^	0.17^∗∗^	–0.18^∗∗^	1	–0.31^∗∗^	–0.06^∗∗^	0.19^∗∗^	–0.09^∗∗^	0.27^∗∗^	–0.11^∗∗^
5. Negative affect	9.72	3.50	–0.04^∗∗^	–0.13^∗∗^	0.27^∗∗^	–0.46^∗∗^	1	0.09^∗∗^	–0.15^∗∗^	0.18^∗∗^	–0.18^∗∗^	0.21^∗∗^
*Caregivers*												
6. Protective buffering	8.86	2.46	0.05^∗∗^	0.05^∗∗^	–0.04^∗∗^	–0.01	0.03^∗^	1	0.25^∗∗^	–0.04^∗∗^	0.09^∗∗^	–0.03
7. Relationship satisfaction	11.87	2.65	0.07^∗∗^	0.18^∗∗^	–0.09^∗∗^	0.12^∗∗^	–0.10^∗∗^	0.23^∗∗^	1	–0.38^∗∗^	0.39^∗∗^	–0.29^∗∗^
8. Relationship stress	1.48	0.81	–0.02	–0.11^∗∗^	0.11^∗∗^	–0.07^∗∗^	0.07^∗∗^	–0.08^∗∗^	–0.29^∗∗^	1	–0.22^∗∗^	0.44^∗∗^
9. Positive affect	15.52	4.57	0.03^∗^	0.06^∗∗^	–0.06^∗∗^	0.14^∗∗^	–0.08^∗∗^	0.11^∗∗^	0.30^∗∗^	–0.25^∗∗^	1	–0.38^∗∗^
10. Negative affect	9.64	3.79	–0.05^∗∗^	–0.08^∗∗^	0.08^∗∗^	–0.13^∗∗^	0.10^∗∗^	–0.09^∗∗^	–0.27^∗∗^	0.34^∗∗^	–0.51^∗∗^	1

*^∗^*p* < 0.05; ^∗∗^*p* < 0.01.*

### Congruence in Protective Buffering Between Dyad Members

**FIGURE 2:**
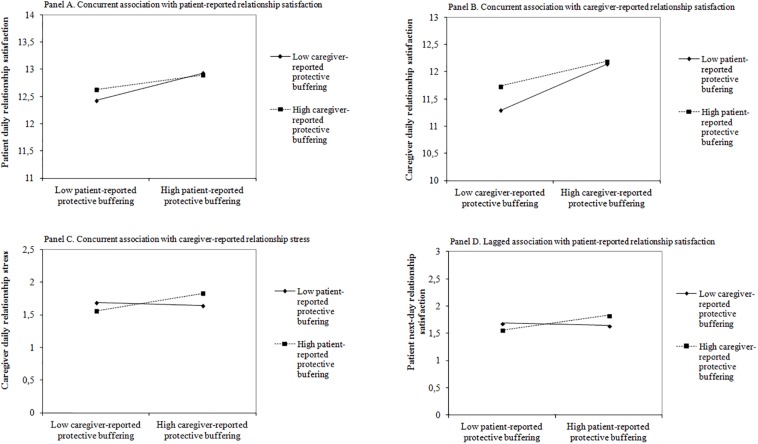
The effect of patient-reported daily protective buffering (PB) × caregiver-reported daily PB on daily relational adjustment indicators. **(A)** Concurrent association with patient-reported relationship satisfaction. **(B)** Concurrent association with caregiver-reported relationship satisfaction. **(C)** Concurrent association with caregiver-reported relationship stress. **(D)** Lagged association with patient-reported relationship satisfaction.

In line with Hypothesis 1, the same-day effect of the interaction between patient- and caregiver-reported daily PB on daily adjustment was significant, although only for its relational indicators (see Table 2). In patients, higher than usual daily PB was related to their higher daily relationship satisfaction regardless of caregivers’ level of daily PB (path a3 in Table 2). Lower than usual patient-reported daily PB was related to their higher daily relationship satisfaction when the caregiver-reported level of daily PB was high (see Figure 2A). A similar beneficial effect of complementarity in daily PB between dyad members was noted for caregiver-reported daily relationship satisfaction (path a4 in Table 2 and Figure 2B) and relationship stress (see Figure 2C).

**TABLE 2:** Results of dyadic MSEM estimating the effect of protective buffering interaction on same-day adjustment indicators (*N* = 200 dyads).

	**Daily relationship satisfaction**		**Daily relationship stress**		**Daily positive affect**		**Daily negative affect**	
				
	**Est. (SE)**	**95% CI**	**Est. (SE)**	**95% CI**	**Est. (SE)**	**95% CI**	**Est. (SE)**	**95% CI**
*Fixed effects (slopes)*								
PB_P_ → ADJ_P_ (*a1*)	0.14(0.03)^***^	[0.07; 0.20]	−0.02(0.01)^*^	[−0.04; −0.003]	0.29(0.07)^***^	[0.16; 0.42]	−0.18(0.08)^*^	[−0.33; −0.03]
PB_C_ → ADJ_P_ (*p1*)	0.03 (0.02)	[−0.01; 0.06]	−0.01(0.01)	[−0.02; 0.001]	0.003 (0.04)	[−0.07; 0.08]	0.02 (0.04)	[−0.05; 0.09]
PB_P_ × PB_C_ → ADJ_P_ (*a3*)	−0.03(0.02)^*^	[−0.06; −0.004]	0.00 (0.01)	[−0.01; 0.01]	−0.04(0.03)	[−0.10; 0.02]	0.02 (0.19)	[−0.35; 0.39]
PB_P_ → ADJ_C_ (*p2*)	0.09(0.03)^***^	[0.04; 0.14]	−0.01(0.01)	[−0.03; 0.004]	0.12(0.05)^*^	[0.03; 0.21]	−0.12(0.04)^**^	[−0.19; −0.05]
PB_C_ → ADJ_C_ (*a2*)	0.23(0.03)^***^	[0.17; 0.29]	−0.04(0.01)^**^	[−0.06; −0.02]	0.27(0.05)^***^	[0.16; 0.38]	−0.18(0.09)^*^	[−0.36; −0.01]
PB_C_ × PB_P_ → ADJ_C_ (*a4*)	−0.05(0.02)^**^	[−0.08; −0.01]	0.02(0.01)^*^	[0.002; 0.03]	−0.03(0.03)	[−0.09; 0.03]	0.05 (0.05)	[−0.04; 0.15]
*Random effects*								
ADJ_P_ variance	1.81(0.18)^***^	[1.45; 2.17]	0.27(0.02)^***^	[0.22; 0.31]	7.17(0.46)^***^	[6.26; 8.08]	4.73(0.48)^***^	[3.89; 5.67]
ADJ_C_ variance	2.36(0.17)^***^	[2.02; 2.70]	0.38(0.02)^***^	[0.33; 0.42]	8.34(0.49)^***^	[7.37; 9.30]	5.67(0.39)^***^	[4.91; 6.42]
ADJ_P_–ADJ_C_ covariance	0.28(0.06)^***^	[0.17; 0.39]	0.03(0.01)^***^	[0.02; 0.05]	0.96(0.18)^***^	[0.62; 1.31]	0.40(0.14)^**^	[0.12; 0.68]
*Model fit*								
Log likelihood	−17,813.27		−8639.52		−23,678.09		−21,937.13	
AIC	35,736.54		17,389.04		47,466.19		43,984.26	
SSABIC	35,917.27		17,569.78		47,646.90		44,165.00	

*P, patients; C, caregiver; PB, protective buffering; ADJ, adjustment indicator, respectively; *a1–a4*, slopes of intrapersonal effects correspond to paths in Figure 1A; *p1–p2*, slopes of interpersonal effect correspond to paths in Figure 1A; AIC, the Akaike information criterion; SSABIC, the sample-size adjusted Bayesian information criterion. ^∗^*p* < 0.05; ^∗∗^*p* < 0.01; ^∗∗∗^*p* < 0.001.*

Contrary to the expectations (Hypothesis 2), daily PB had concurrent individual and relational benefits for both patients and caregivers (see Table 2). Patient- and caregiver-reported higher than usual daily PB were associated with their own better adjustment, i.e., higher same-day relationship satisfaction and PA and lower same-day relationship stress and NA (paths a1 and a2). Also, we noted a significant beneficial interpersonal effect from patient-reported daily PB to caregiver-reported daily adjustment (path p2). Higher than usual patient daily PB was related to higher same-day relationship satisfaction and PA, as well as lower same-day NA in caregivers.

In terms of the lagged effects of dyad member daily PB on daily adjustment, only significant interaction slope for daily relationship satisfaction was noted (see Table 3, path a3). This time, contrary to Hypothesis 1, similarity in daily PB between dyad members was beneficial (see Figure 2D). Higher patient relationship satisfaction was related to similarity in the previous-day level of PB in patients and caregivers.

**TABLE 3:** Results of dyadic MSEM estimating the effect of protective buffering interaction on next-day adjustment indicators (*N* = 200 dyads).

	**Daily relationship satisfaction**		**Daily relationship stress**		**Daily positive affect**		**Daily negative affect**	
				
	**Est. (SE)**	**95% CI**	**Est. (SE)**	**95% CI**	**Est. (SE)**	**95% CI**	**Est. (SE)**	**95% CI**
*Fixed effects (slopes)^1^*								
PB_P_ → ADJ_P_ (*a1*)	0.04 (0.02)	[0.00; 0.09]	−0.02(0.01)	[−0.04; 0.01]	0.03 (0.06)	[−0.08; 0.14]	−0.03(0.03)	[−0.09; 0.04]
PB_C_ → ADJ_P_ (*p1*)	0.01 (0.02)	[−0.02; 0.04]	0.00 (0.01)	[−0.02; 0.02]	−0.05(0.05)	[−0.14; 0.04]	0.01 (0.02)	[−0.03; 0.05]
PB_P_ × PB_C_ → ADJ_P_ (*a3*)	0.04(0.01)^*^	[0.01; 0.06]	−0.00(0.01)	[−0.03; 0.02]	0.02 (0.04)	[−0.05; 0.10]	−0.01(0.02)	[−0.05; 0.03]
PB_P_ → ADJ_C_ (*p2*)	0.01 (0.02)	[−0.04; 0.06]	−0.01(0.02)	[−0.04; 0.02]	0.03 (0.06)	[−0.08; 0.15]	−0.05(0.03)	[−0.12; 0.01]
PB_C_ → ADJ_C_ (*a2*)	0.04(0.02)^*^	[0.004; 0.08]	−0.01(0.02)	[−0.04; 0.01]	0.02 (0.06)	[−0.10; 0.14]	−0.04(0.03)	[−0.10; 0.03]
PB_C_ × PB_P_ → ADJ_C_ (*a4*)	−0.02(0.02)	[−0.05; 0.02]	0.00 (0.01)	[−0.02; 0.02]	0.00 (0.04)	[−0.08; 0.08]	0.00 (0.03)	[−0.05; 0.05]
*Random effects*								
ADJ_P_ variance	1.68(0.15)^***^	[1.39; 1.98]	0.25(0.01)^***^	[0.23; 0.26]	6.30(0.24)^***^	[5.84; 6.76]	4.44(0.36)^***^	[3.73; 5.15]
ADJ_C_ variance	2.31(0.16)^***^	[1.99; 2.63]	0.37(0.01)^***^	[0.35; 0.40]	8.11(0.34)^***^	[7.44; 8.78]	5.56(0.38)^***^	[4.82; 6.30]
ADJ_P_–ADJ_C_ covariance	0.27(0.05)^***^	[0.17; 0.27]	0.03(0.02)^*^	[0.01; 0.06]	0.81(0.28)^**^	[0.26; 1.36]	0.43(0.11)^***^	[0.21; 0.65]
*Model fit*								
Log likelihood	−33,528.62		−15,799.236		−44,298.08		−41,251.40	
AIC	67,185.24		31,726.472		88,724.15		82,630.81	
SSABIC	67,393.45		31,934.687		88,932.37		82,839.02	

*P, patients; C, caregiver; PB, protective buffering; ADJ, adjustment indicator, respectively; *a1–a4*, slopes of intrapersonal effects correspond to paths in Figure 1A; *p1–p2*, slopes of interpersonal effect correspond to paths in Figure 1A; AIC, the Akaike information criterion; *SSABIC*, the sample-size adjusted Bayesian information criterion. ^∗^*p* < 0.05; ^∗∗^*p* < 0.01; ^∗∗∗^*p* < 0.001. ^1^Controlled for the previous day outcome.*

Also, one caregiver intrapersonal effect was significant indicating that the positive effect of caregiver-reported daily PB on caregiver daily relationship satisfaction had also next-day extended effect (path a2 in Table 3). We found no other lagged associations between daily PB and adjustment in dyads following HSCT.

All concurrent models fit the data better than the lagged ones. All MSEM models were repeated with covariates which had been significantly related to intercept and/or slope of dyad member daily adjustment indicators in preliminary. The inclusion of these variables did not alter the findings. Thus, models without covariates were presented for parsimony.

### Reciprocal Dependency in Relationship Between Protective Buffering and Adjustment

**FIGURE 3:**
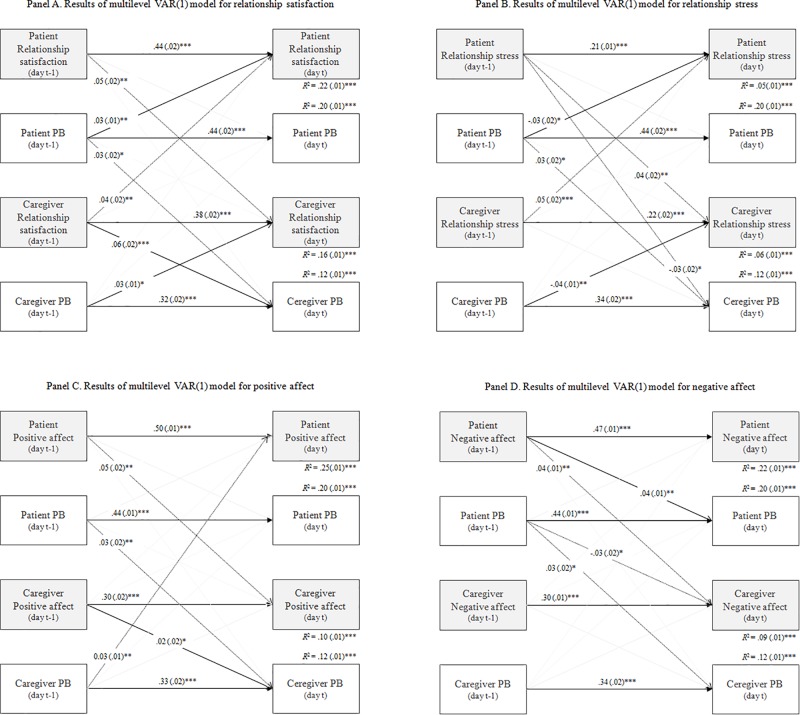
Autoregressive cross-lagged model results. **(A)** Results for daily relationship satisfaction. **(B)** Results for daily relationship stress. **(C)** Results for daily positive affect. **(D)** Results for daily negative affect. Significant standardized coefficients (controlled for the within-person variance) are presented; *Estimation* (Posterior mean). Covariances are omitted. ^∗^*p* < 0.05; ^∗∗^*p* < 0.01; ^∗∗∗^*p* < 0.001.

Table 4 shows row estimates (posterior means) for fixed and random effects and Figure 3 shows significant standardized estimates for fixed effects in autoregressive cross-lagged models. In line with Hypothesis 3 and regardless of the adjustment indicator, autoregressive paths within dyad members were the strongest (paths a1, a2, and a5, a6 in Figure 1 and Table 4). The average autoregressive parameters cross dyad members (paths p1, p4, and p5, p8) were rather small but consistently suggested the interpersonal effect from patients (adjustment, PB) to caregivers (adjustment, PB, respectively), especially for individual adjustment indicators (Figures 3C,D). For relational adjustment indicators (Figures 3A,B), an additional path of the same magnitude emerged from caregiver-reported to patient-reported adjustment as described above.

**TABLE 4:** Results of multilevel VAR(1) models estimating the autoregressive and cross-lagged effects between daily protective buffering and adjustment indicators (row ratings).

	**Daily relationship satisfaction**		**Daily relationship stress**		**Daily positive affect**		**Daily negative affect**	
				
	**Est.**	**95% CI**	**Est.**	**95% CI**	**Est.**	**95% CI**	**Est.**	**95% CI**
*Fixed effects*								
ADJ_P_ (*t* − 1) → ADJ_P_ (*a1*)	0.44(0.01)^***^	[0.41; 0.47]	0.21(0.01)^***^	[0.18; 0.24]	0.50(0.01)^***^	[0.47; 0.53]	0.47(0.01)^***^	[0.44; 0.50]
PB_P_ (*t* − 1) → PB_P_ (*a2*)	0.44(0.01)^***^	[0.41; 0.47]	0.44(0.01)^***^	[0.41; 0.47]	0.44(0.01)^***^	[0.41; 0.47]	0.44(0.01)^***^	[0.41; 0.47]
ADJ_P_ (*t* − 1) → PB_P_ (*a4*)	0.02 (0.01)	[−0.01; 0.04]	0.01 (0.04)	[−0.06; 0.08]	0.00 (0.01)	[−0.01; 0.01]	0.02(0.01)^**^	[0.01; 0.04]
PB_P_ (*t* − 1) → ADJ_P_ (*a3*)	0.03(0.01)^**^	[0.01; 0.06]	−0.01(0.01)^*^	[−0.02; 0.00]	0.00 (0.03)	[−0.06; 0.06]	0.02 (0.02)	[−0.02; 0.07]
ADJ_C_ (*t* − 1) → ADJ_C_ (*a5*)	0.38(0.01)^***^	[0.35; 0.40]	0.22(0.02)^***^	[0.19; 0.25]	0.30(0.01)^***^	[0.27; 0.33]	0.30(0.01)^***^	[0.27; 0.33]
PB_C_ (*t* − 1) → PB_C_ (*a6*)	0.32(0.01)^***^	[0.29; 0.35]	0.33(0.01)^***^	[0.30; 0.36]	0.33(0.01)^***^	[0.30; 0.36]	0.33(0.01)^***^	[0.30; 0.36]
ADJ_C_ (*t* − 1) → PB_C_ (*a7*)	0.05(0.01)^***^	[0.03; 0.08]	−0.04(0.03)	[−0.10; 0.03]	0.01 (0.01)	[−0.002; 0.03]	−0.01(0.01)	[−0.03; 0.01]
PB_C_ (*t* − 1) → ADJ_C_ (*a8*)	0.03(0.02)^*^	[0.002; 0.07]	−0.02(0.01)^**^	[−0.03; −0.01]	0.04 (0.03)	[−0.02; 0.10]	−0.02(0.02)	[−0.07; 0.03]
ADJ_P_ (*t* − 1) → ADJ_C_ (*p1*)	0.06(0.02)^**^	[0.02; 0.09]	0.05(0.02)^**^	[0.01; 0.08]	0.05(0.02)^**^	[0.02; 0.08]	0.04(0.01)^**^	[0.01; 0.07]
ADJ_P_ (*t* − 1) → PB_C_ (*p2*)	0.02 (0.01)	[−0.01; 0.05]	−0.07(0.04)^*^	[−0.15; 0.00]	−0.01(0.01)	[−0.03; 0.003]	0.01 (0.01)	[−0.01; 0.02]
PB_P_ (*t* − 1) → ADJ_C_ (*p3*)	0.03 (0.02)	[−0.01; 0.06]	−0.01(0.01)	[−0.02; 0.00]	0.03 (0.03)	[−0.03; 0.09]	−0.04(0.03)	[−0.10; 0.01]
PB_P_ (*t* − 1) → PB_C_ (*p4*)	0.03(0.01)^*^	[0.00; 0.06]	0.03(0.01)^*^	[0.004; 0.06]	0.04(0.01)^**^	[0.01; 0.07]	0.03(0.01)^*^	[0.01; 0.06]
ADJ_C_ (*t* − 1) → ADJ_P_ (*p5*)	0.04(0.01)^**^	[0.01; 0.06]	0.04(0.01)^***^	[0.02; 0.07]	0.00 (0.01)	[−0.02; 0.03]	0.00 (0.01)	[−0.02; 0.02]
ADJ_C_ (*t* − 1) → PB_P_ (*p6*)	0.00 (0.01)	[−0.02; 0.03]	−0.01(0.03)	[−0.07; 0.05]	0.00 (0.01)	[−0.02; 0.01]	−0.01(0.01)	[−0.02; 0.01]
PB_C_ (*t* − 1) → ADJ_P_ (*p7*)	0.01 (0.01)	[−0.02; 0.04]	−0.00(0.00)	[−0.01; 0.01]	−0.07(0.03)^**^	[−0.12; −0.01]	0.03 (0.02)	[−0.01; 0.08]
PB_C_ (*t* − 1) → PB_P_ (*p8*)	0.02 (0.01)	[−0.01; 0.05]	0.02 (0.01)	[−0.01; 0.05]	0.02 (0.01)	[−0.01; 0.05]	0.02 (0.01)	[−0.01; 0.04]
*Random effects*								
ADJ_P_ variance	1.93(0.04)^***^	[1.86; 2.01]	0.28(0.01)^***^	[0.27; 0.29]	6.80(0.14)^***^	[6.55; 7.09]	4.87(0.10)^***^	[4.68; 5.07]
ADJ_C_ variance	2.61(0.05)^***^	[2.51; 2.71]	0.41(0.01)^***^	[0.39; 0.42]	8.71(0.18)^***^	[8.36; 9.07]	5.96(0.12)^***^	[5.73; 6.20]
PB_P_ variance	1.70(0.03)^***^	[1.63; 1.77]	1.70(0.04)^***^	[1.63; 1.77]	1.70(0.03)^***^	[1.63; 1.77]	1.70(0.03)^***^	[1.63; 1.77]
PB_C_ variance	1.91(0.04)^***^	[1.84; 1.99]	1.92(0.04)^***^	[1.84; 2.00]	1.92(0.04)^***^	[1.84; 2.00]	1.92(0.04)^***^	[1.84; 2.00]
ADJ_P_–ADJ_C_ covariance	0.35(0.03)^***^	[0.28; 0.41]	0.03(0.01)^***^	[0.02; 0.04]	0.96(0.11)^***^	[0.73; 1.18]	0.48(0.08)^***^	[0.32; 0.63]
ADJ_P_–PB_P_ covariance	0.30(0.03)^***^	[0.25; 0.35]	−0.03(0.01)^**^	[−0.05; −0.01]	0.36(0.05)^***^	[0.26; 0.46]	−0.18(0.04)^***^	[−0.26; −0.10]
ADJ_P_–PB_C_ covariance	0.07(0.03)^*^	[0.01; 0.13]	−0.02(0.01)^*^	[−0.04; 0.00]	0.00 (0.05)	[−0.11; 0.10]	0.06 (0.05)	[−0.03; 0.15]
ADJ_C_–PB_P_ covariance	0.13(0.03)^***^	[0.07; 0.20]	−0.01(0.01)	[−0.04; 0.01]	0.12(0.06)^*^	[0.003; 0.23]	−0.12(0.05)^**^	[−0.21; −0.02]
ADJ_C_–PB_C_ covariance	0.44(0.03)^***^	[0.37; 0.50]	−0.05(0.01)^***^	[−0.08; −0.03]	0.41(0.06)^***^	[0.30; 0.54]	−0.28(0.05)^***^	[−0.37; −0.18]
PB_P_–PB_C_ covariance	0.06(0.03)^*^	[0.004; 0.11]	0.05(0.03)^*^	[0.003; 0.11]	0.06(0.03)^*^	[0.002; 0.11]	0.06(0.03)^*^	[0.003; 0.11]
*Model fit*								
DIC	1,21,072.23		1,00,069.31		1,35,216.60		1,31,249.16	

*P, patients; C, caregiver; PB, protective buffering; ADJ, adjustment indicator, respectively; *a1–p8*, slopes correspond to paths in Figure 1B; DIC, the deviance information criterion. ^∗^*p* < 0.05; ^∗∗^*p* < 0.01; ^∗∗∗^*p* < 0.001.*

The inspection of the cross-lagged parameters indicated that the 1-day causal effects differed for both patients and caregivers (paths a3, a4, a8, a8, and pa2, p3, p6, p7). For patients, the picture was more consistent across adjustment indicators pointing to own or caregiver-reported daily PB to next-day adjustment effects (Figures 3A–C). However, for NA the reverse relationship was found, i.e., from patient’s affect to next-day PB (Figure 3D). For caregivers, we noted similar results for positive adjustment indicators suggesting the adjustment to next-day PB effects (Figures 3A,C). For negative indicators, the reverse associations seem to be noted (Figures 3B,D; the exception was the significant negative path from patient-reported relationship stress in *t* − 1 to caregiver-reported PB in day *t*).

The averaged within-person proportion of explained variance was the lowest for relationship stress, and higher for NA, PA and relationship satisfaction. Generally, the parameters were stronger for patients than caregivers. All DSEM models were repeated with covariates (besides significant confounders for adjustment indicators, patient age, sex, and education, patient and caregiver employment, relationship duration and type of transplant were included as they were significantly related to intercept or slope of participants’ PB). The inclusion of these variables did not alter the findings. For parsimony, models without confounders were presented.

## Discussion

The study aimed at examining within-person associations between patient and caregiver PB and positive and negative both individual- and dyad-level adjustment to HSCT. In particular, we investigated (1) the congruence in daily PB (Hypothesis 1), (2) the advantage of PB for relational not individual adjustment (Hypothesis 2), and (3) the reciprocal associations in daily PB-adjustment relationship (Hypothesis 3). To the best of our knowledge, this study is the first to investigate these issues.

In line with Hypothesis 1 and a previous study (Badr, 2004), complementarity in daily PB was related to better same-day relational adjustment, i.e., higher relationship satisfaction in patients and caregivers as well as lower relationship stress in caregivers. For positive relational adjustment, complementarity was of particular importance in the case of participant lower than usual daily PB. A slightly different complementarity was observed for negative relational adjustment in caregivers. Complementarity between caregiver- and patient-reported daily PB was associated with caregivers’ lower daily relationship stress regardless of caregiver’s PB level. It can indicate a slightly different mechanism of evoking positive and negative relational adjustment. Generally, this finding suggests the benefits of daily PB in our sample indicating that the situation when one of the dyad members protects the other is sufficient for better relational adjustment in dyads after HSCT. This may result from the stereotypical perception of a person who “copes well” in a difficult situation as a controlled individual that does not reveal emotions or anxiety. “Being strong” alone or having a “strong” partner apparently favored a better assessment of the relationship in our group. The above relationships were correlative only. However, lagged analyses reflecting short-time predictions did not confirm these assumptions. Similarity not complementarity in patient–caregiver daily PB predicted higher next-day relationship satisfaction but only in patients. Similarity between dyad members in supportive communication probably results in more predictable and therefore enjoyable communication (Berger and Calabrese, 1975). It most probably involves a greater sense of mutual understanding, closeness and consequently a better assessment of the relationship. Previous studies suggested that similarity is especially effective for the so-called adaptive behaviors (Revenson, 1994; Revenson and DeLongis, 2011). In our case, the function of PB was adaptive. Therefore its lagged beneficial effect should not be surprising.

The findings did not support Hypothesis 2. Daily PB has positive effects in patient–caregiver dyads following HSCT, especially in concurrent analyses (for both relational and individual adjustment, both positive and negative). This result supports the inconclusive data indicating both favorable (Coyne and Smith, 1994; Finkenauer and Hazam, 2000; Bonanno et al., 2004; Liverant et al., 2008; Roberts et al., 2008) and unfavorable effects of PB (Coyne and Smith, 1991; Suls et al., 1997; Butler et al., 2004; Hinnen et al., 2007, 2009; Langer et al., 2009, 2018; Manne et al., 2010; Vilchinsky et al., 2011). Benefits of daily PB concern mainly intrapersonal effects. Advantages of daily PB in individuals who use it may result from experiencing a sense of control or self-efficacy on the one hand, as already demonstrated by Coyne and Smith (1991). On the other hand, they may be associated with a sense of fulfilling the adopted role (patient, caregiver) and the role-related social norms (see e.g., Glajchen, 2004). The reported interpersonal effects from patient daily PB to caregiver greater adjustment (including relationship satisfaction and PA and NA) may support this hypothesis. Caregivers reported a better adaptation on the days when patients did not reveal their fears or negative emotions or when they did not report complaints. From the perspective of the caregiver, such behavior could be perceived as an expression of good mental well-being of the patient and no physical complaints. Potentially, it could also be reciprocally perceived as proper fulfillment of one’s role in this process. Perhaps our participants were driven by different motivation to PB, i.e., in the case of patients it was willingness to protect the partner whereas for caregivers it was willingness to protect themselves from stress. Langer et al. (2009) confirmed that in patients following HSCT and their caregivers prosocial motivation to protect a partner had a delayed, 50-day association with higher relationship satisfaction in patients while in caregivers an egoistic motivation was related to better relationship adjustment. In terms of the delayed effects in our study, only the beneficial intrapersonal effect for relationship satisfaction of caregivers was present the following day. It also showed that daily PB is associated with relational and individual adjustment by correlative (same-day) rather than predictive (next-day) relationships.

Analyses examining reciprocal relationships (causality) between these variables supported these assumptions. In accordance with Hypothesis 3, fluctuation in daily PB and adjustment indicators were characterized mainly by regulatory weakness, i.e., resistance to change from day to day as a result of external (partner’s behavior) or internal events (one’s own behavior). For models with individual adjustment, autoregressive effect emerged also across dyad members. It may result from the fact that the patient is in the center of attention following HSCT, and in a way “controls” the situation at home, i.e., his/her behavior and emotions are transferred to family members (Neumann and Strack, 2000), including the individual adaptation of the closest caregivers. For dyad-level adjustment indicators, the autoregressive effects occurred in both directions, i.e., from the patient to the caregiver but also from the caregiver to the patient. Thus, both sides of the interaction participated in shaping relational indicators, which seems to be in line with the origin of this form of adaptation.

Cross regression effects were distinct for patients and caregivers as well as for positive and negative (but not relational *versus* individual) adjustment indicators. For patients, daily PB to next-day adjustment effects were noted except for NA. Thus, supportive communication regulated emotional adaptation to HSCT, confirming relationships described in the transactional stress and coping model (Lazarus and Folkman, 1984) as well as the causal model of social support and health (Schwarzer and Leppin, 1991). For caregivers, a similar pattern was observed for negative adjustment indicators only. We found opposite effects (from daily adjustment to PB) for positive adjustment (both individual and relational). Positive indicators were the driving force behind behavior, which is in line with the broaden-and-build theory of positive emotions (Fredrickson, 2001).

From a practical point of view, clinicians should be conscious that PB-related activities can also bring benefits to individuals and dyads in respect to different levels of adjustment to the disease. We recommend focusing on congruence (whether dyad members are similar or complementary in PB) and recognizing the preferences and motivations related to PB of both sides. Learning mutual expectations by both partners related to the level of openness of supportive communication and its extent may improve its effectiveness, contributing to better adaptation. Also, the awareness of partners that both emotional and behavioral processes tend to “lag” may protect against disappointment and a sense of helplessness when attempts to improve mood or change in behavior (one’s own or partner’s) do not produce quick results. In turn, awareness that both sides participate in shaping relational adaptation and that patient behavior and emotions are transferred to family members can increase self-awareness of the causes of their own mental processes and contribute to increased mindfulness in the relationship.

Our study has also some limitations. First, patient–caregiver dyads following HSCT may be a specific group in terms of analyzing PB effects, especially immediately after discharge. HSCT is a life-threatening procedure and both sides are aware of it. In addition, during hospitalization, the patient must be isolated and visits are not allowed, which results in the fact that for at least 3 weeks (if not much longer in the case of allogeneic HSCT recipients), patients have no direct contact with their relatives. These factors may affect the mutual contacts and their assessment in the post-discharge period. However, the aim of the study was to last for a month to allow the subjects to “return” to their baseline interaction patterns prior to HSCT. Secondly, our group was heterogeneous in terms of the time that elapsed from the diagnosis and initiating the process related to coping with the disease (from 3 months to 15 years). It cannot be ruled out that the effectiveness of PB is subject to certain changes over time and that dyads learn the most beneficial forms of mutual communication and support with the disease duration. Therefore, it is possible that we obtained different results compared to previous studies, which included patients during a short period following the diagnosis. Thirdly, our sample was heterogeneous in terms of the type of relationship between patients and caregivers. Future studies on the role of daily PB in spousal and non-spousal dyads are needed. Finally, lagged effects were limited to one-day effects. It should be borne in mind when the results are interpreted or possibly generalized. Further studies should consider a longer period of time (i.e., two- or three-day effects) to confirm the directions of relationships between PB fluctuation and adjustment in dyads.

Despite the limitations, our findings suggest that the effect of daily PB in dyads following HSCT depends on support timing (same- or next-day effect) and is different for both parties. We found no costs from protection of the partner or relationship against revealing negative states in dyads following HSCT. Patients seemed to have benefited the most from the similarity in daily PB, while caregivers profited from complementarity. Causal associations between PB and adjustment were also different and opposite in patients and caregivers.

## Data Availability Statement

The datasets for this manuscript are not publicly available because the data underlying this study contain potentially identifying information (as comes from two related persons) and cannot be publicly shared. Requests to access the datasets should be directed to SWPS University of Social Sciences and Humanities, Faculty of Psychology Ethics Committee, psychoetyka@swps.edu.pl.

## Ethics Statement

The study was conducted in accordance with the recommendations of the SWPS University of Social Sciences and Humanities Ethics Committee and the University Ethics Committee approved the protocol (decision no. 24/2014). All participants gave written informed consent in accordance with the Declaration of Helsinki. Participation in the study was voluntary.

## Author Contributions

AK contributed to the conception, design, acquisition, analyzed, and interpretation of the data for the work. AK and MS-K drafted the manuscript and revised it critically for important intellectual content, approved the final version to be published, and agreed to be accountable for all aspects of the work in ensuring that questions related to the accuracy or integrity of any part of the work are appropriately investigated and resolved.

## Conflict of Interest

The authors declare that the research was conducted in the absence of any commercial or financial relationships that could be construed as a potential conflict of interest.
